# Radiation-Induced Uterine Carcinosarcoma after Concurrent Chemoradiotherapy for Cervical Squamous Cell Carcinoma

**DOI:** 10.1055/s-0038-1673678

**Published:** 2018-10-11

**Authors:** Joana Moreira-Barros, Kuan-Gen Huang, Tsung-Hsun Tsai

**Affiliations:** 1 Department of Obstetrics and Gynecology, Hospital Pedro Hispano, Matosinhos, Portugal; 2 Department of Obstetrics and Gynecology, Chang Gung Memorial Hospital, Linkou Medical Center, Taoyuan, Taiwan

**Keywords:** carcinosarcoma, carcinogenesis, cervical cancer, radiation-induced neoplasms, uterine neoplasms

## Abstract

**Objective** To describe a case of radiation-induced uterine carcinosarcoma 6 years after a cervical squamous cell carcinoma treatment, which imposed some diagnostic and management challenges.

**Case Report** A 57-year-old woman with a history of pelvic chemoradiotherapy ∼ 6.5 years before the event described in this study, following an International Federation of Gynecology and Obstetrics (FIGO) stage IIB cervical cancer, presented with a cervical mass, involving the uterine cavity, the cervical canal and the upper two thirds of the vagina. The biopsy showed a poorly differentiated carcinoma, and a positron emission tomography (PET) scan excluded distant metastasis, although it was unable to define the origin of the tumor as either a new primary malignancy of the endometrium/cervix or as a cervical recurrence. Surgical staging procedure was performed, and the diagnosis was endometrial carcinosarcoma, FIGO stage IIB. The patient was not able to complete the adjuvant therapy, and the progression of the disease was remarkable.

**Conclusion** The present case highlights one of the less common but more serious consequences of radiotherapy for cervical cancer, which has an increasing incidence in younger women, raising concerns about the long-term consequences of its management.

## Introduction

Uterine carcinosarcoma (UCS), also known as malignant mixed mesodermal tumor or malignant mixed Müllerian tumor, is an uncommon and very aggressive uterine neoplasm, responsible for < 5% of all uterine malignancies and with a mortality rate of ∼ 15%.^1^ ^2^ Composed of epithelial and mesenchymal malignant elements, UCSs usually occur in postmenopausal women, with a peak incidence between the ages of 60 and 70 years old.^1^ ^3^ Previously, it was considered that UCSs behaved like sarcomas, but recent molecular and genetic data have suggested that the epithelial component is the major driver of tumor aggressiveness, and these rare neoplasms are now grouped with the endometrial carcinomas and are treated the same way as poorly differentiated adenocarcinomas.^1^ ^4^ It is thought that the origin of the tumor follows the embryological development path of the Müllerian ducts and is most likely derived from a pluripotent stem cell that differentiates into both epithelial and mesenchymal cell types. Further characterization of the tumor histology supports the “conversion” theory, which states that an epithelial-to-mesenchymal (EMT) transition occurs because the sarcomatous portion of the tumor exhibits markers consistent with an epithelial origin.^5^ Integrated genomic, epigenomic, transcriptomic, and proteomic analysis of UCSs identified multiple somatic mutations and copy-number alterations that offer expanded therapeutic options, including potential use of poly adenosine diphosphate-ribose polymerase (PARP), enhancer of zeste homolog 2 (EZH2), cell-cycle, and phosphoinositide 3-kinase (PI3K) pathway inhibitors. The EMT features recognized in UCSs also provide a mechanistic basis for the conversion of serous-like endometrial carcinoma precursors in a majority of cases.^5^ ^6^ Known risk factors for the development of UCSs are increased estrogen levels (especially exogenous), obesity, nulliparity, ethnicity (black women), prolonged selective estrogen-receptor modulator (tamoxifen) use and history of pelvic radiation.^1^ ^2^ ^3^ Radiation therapy—usually with radiosensitizing chemotherapy—is the main treatment for locally advanced cervical cancer, being associated with many adverse effects, especially long-term complications. Secondary malignancy of pelvic organs is an uncommon, but important concern of radiation therapy for cervical cancer.^7^ About 15% of the cases of previous pelvic radiation are estimated to progress to UCS, mostly after malignancies of the uterine cervix.^1^ ^3^ Cervical cancer is the fourth most common cancer in women, and the seventh overall.^7^ Epidemiological reports suggest comparatively declining trends in cervical cancer incidence and mortality rates in Taiwan and other Asian countries, however, with increasing incidence of cases in young women.^8^ We describe a case of UCS diagnosed 6 years after a stage IIB cervical cancer managed with chemoradiation therapy, in a 57-year-old woman, which imposed some diagnostic and management challenges, ending up in a tragic outcome, characteristic of these aggressive neoplasms.

## Case Report

**Fig. 1:**
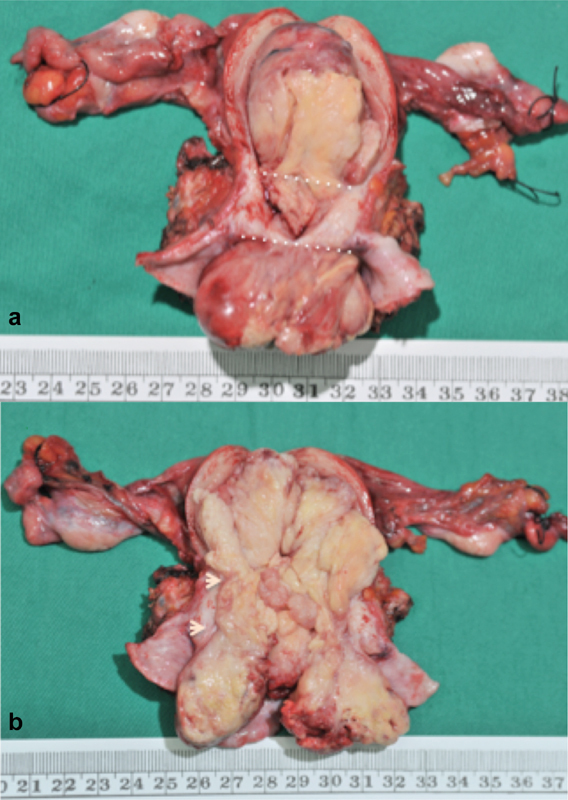
**(a)-(b)** Solid tumor occupying the entire uterine cavity, the uterine cervix, and the upper two thirds of the vagina; the landmarks indicate internal cervical os and external cervical os level.

**Fig. 2:**
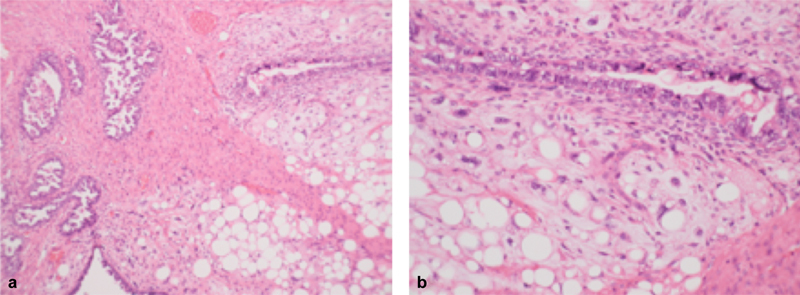
Pathologic specimen of carcinosarcoma—(a) biphasic tumor with carcinomatous and sarcoma-like elements (hematoxylin-eosin,10x); (b) heterologous type sarcoma with lipomatous differentiation and epithelial component—neoplastic glands—are present (hematoxylin-eosin, 20x).

A 50-year-old woman, 2G2P (2 cesarean sections) with chronic hypertension and type II diabetes mellitus, sought medical care in January 2007 reporting postintercourse bleeding for 1 year, without other complaints. On speculum investigation, an exophytic tumor of the cervix, ∼ 7 cm, was found, and the pelvic examination showed a cervical mass with posterior parametrium invasion. After a complete investigation, the patient was diagnosed with cervical squamous cell carcinoma, International Federation of Gynecology and Obstetrics (FIGO) stage IIB (cT2bN1Mx). She completed the radiation and chemotherapy treatment (whole pelvis radiation up to 7020 cGy and 6 cycles of cisplatin [60mg/m^2^]), achieving tumor regression, without reported complications. Pelvic examination, cervical smear, computed tomography (CT), and magnetic resonance imaging (MRI) exams were performed regularly thereafter, without evidence of disease recurrence during the 5-year follow-up. Eighteen months later, and ∼ 6.5 years after the radiation therapy, the patient presented to the emergency department complaining of vaginal spotting for 2 weeks. The gynecologic examination revealed a 5 cm cervical mass. A biopsy was performed, which was consistent with a poorly differentiated carcinoma. An MRI exam and a PET scan showed a large soft tissue mass occupying the entire uterine cavity, the uterine cervix and the upper two thirds of the vagina, with no definite evidence of lymph node or distant involvement. The origin of the primary tumor was imprecise, and the diagnostic hypotheses of tumor recurrence, endocervical/endometrial cancer *de novo*, namely carcinosarcoma, were proposed. Accordingly, the patient completed the surgical staging with total abdominal hysterectomy, bilateral salpingo-oophorectomy (Fig. 1a and 1b), pelvic and para-aortic lymphadenectomy, and peritoneal washing for cytology. The final pathologic report was consistent with the diagnosis of endometrial carcinosarcoma, pT2bN0M0, FIGO stage IIB (Fig. 2a and 2b). The patient was not able to complete the adjuvant therapy, and the progression of the disease was remarkable. The patient died due to urosepsis 16 months after the diagnosis.

## Discussion

Previous exposure to radiation is the most commonly associated etiological factor of carcinosarcoma,^9^ particularly after radiotherapy for malignancies of the uterine cervix,^3^ as in the present case. Two mechanisms were proposed for the pathogenesis of radiation-induced malignancies: (a) radiation: single and double strand DNA breaks, which lead to gene mutation and malignant transformation of the radiated cells; and (b) the bystander or abscopal effect: the inflammatory cytokines released from the radiated cells induce increased reactive oxygen species, causing DNA damage.^10^ The patient developed UCS 6 years after the radiation therapy for cervical cancer, with a total pelvic radiation dose of 7,020 cGy. In the previously published cases, the interval between the time of radiation and the diagnosis of UCS varies between 3 and 30 years, with the total radiation doses ranging from 2,400 to 8,000 cGy, and the most common type of secondary uterine malignancy is carcinosarcoma.^3^ ^4^ ^10^ No specific clinical or histopathological features distinguish between radiation-associated and *de novo* uterine carcinosarcomas,^3^ often making the diagnosis challenging. In the described patient, the size of the tumor, added to the cervical, endometrial and parametric involvement and to the unspecific histology, hampered the diagnosis, raising the hypothesis of a recurrence of cervical cancer or of a primary cervical/endometrial cancer *de novo*. There are some criteria to consider a carcinosarcoma to be radiation-associated, including: (i) its development must be within a previously irradiated field; (ii) patients should have received a significant amount of radiation; (iii) a latency period of several years (at least 3–5 years) must elapse between the time of radiation and the development of the malignancy; (iv) the diagnosis must be histologically proven; and (v) the secondary tumor should be histologically different from the primary neoplasm.^4^ ^10^ Our patient met all the criteria listed. The initial treatment recommended for early-stage UCS is surgical staging, defined as hysterectomy, bilateral salpingo-oophorectomy, and systematic lymphadenectomy. Cytoreductive surgery with complete gross resection should be considered for advanced-stage UCS cases, which was found to improve the overall survival. Data on adjuvant chemotherapy and/or radiation therapy are limited and often conflicting, although the 2016 National Comprehensive Cancer Network (NCCN) guidelines recommend adjuvant treatment in most stages of UCS.^1^ ^4^ ^9^ Future genetic characterization may offer the potential to increase the therapeutic options, improving the prognosis of this rare type of cancer.^5^ ^6^ In the present case, the patient was not able to complete the adjuvant therapy and the progression of the disease was remarkable. In conclusion, the present case highlights one of the less common but more severe consequences of radiotherapy. Although rare, UCS is a very aggressive uterine neoplasm, and found more frequently after radiation therapy for cervical cancer. While the overall incidence of UCS appears to be declining, the incidence of cervical cancer in young women is increasing, rising concerns about long-term consequences of its management.
